# The Effects of Adolescent Childbearing on Literacy and Numeracy in Bangladesh, Malawi, and Zambia

**DOI:** 10.1007/s13524-019-00816-z

**Published:** 2019-09-09

**Authors:** Stephanie R. Psaki, Erica Soler-Hampejsek, Jyotirmoy Saha, Barbara S. Mensch, Sajeda Amin

**Affiliations:** 1 Population Council, One Dag Hammarskjold Plaza, New York, NY 10017 USA; 2 Madrid, Spain; 3 Population Council, Dhaka, Bangladesh

**Keywords:** Adolescent childbearing, Literacy, Numeracy, Education, Longitudinal data

## Abstract

Global investments in girls’ education have been motivated, in part, by an expectation that more-educated women will have smaller and healthier families. However, in many low- and middle-income countries, the timing of school dropout and first birth coincide, resulting in a rapid transition from the role of student to the role of mother for adolescent girls. Despite growing interest in the effects of pregnancy on levels of school dropout, researchers have largely overlooked the potential effect of adolescent childbearing on literacy and numeracy. We hypothesize that becoming a mother soon after leaving school may cause the deterioration of skills gained in school. Using longitudinal data from Bangladesh, Malawi, and Zambia, we test our hypothesis by estimating fixed-effects linear regression models to address the endogeneity in the relationship between childbearing and academic skills. To our knowledge, this is the first study to examine the effects of adolescent childbearing on academic skills in low- and middle-income countries. Our results indicate that among those with low levels of grade attainment, first birth has a negative effect on English literacy and numeracy. Among those with higher levels of grade attainment, we find little evidence of effects of childbearing on academic skills. Childbearing also has little effect on local language literacy. Beyond the immediate loss of English literacy and numeracy, if these skills lead to better health and more economic productivity, then adolescent childbearing may have longer-term repercussions than previously understood. In addition to ongoing efforts to increase educational attainment and school quality in low- and middle-income countries, investments are needed to strengthen the academic skills of adolescent mothers to secure the demographic and economic promise of expanded education for girls and women.

**Electronic supplementary material:**

The online version of this article (10.1007/s13524-019-00816-z) contains supplementary material, which is available to authorized users.

## Introduction

Investments in girls’ education globally have been motivated in part by an expectation that more-educated women will have smaller and healthier families (Behrman 2015; Grant 2015; Psaki et al. 2019; Weitzman 2017). However, despite dramatic gains in women’s attainment globally, the pace of fertility decline is slower than expected in some countries (Bongaarts and Casterline 2013; Nguyen and Wodon 2012), raising the question, under which conditions is women’s education most likely to translate into improved demographic outcomes?

Some researchers have speculated that academic skills are the primary pathway through which grade attainment influences fertility (LeVine et al. 2012). Literacy may improve access to information about the benefits of birth spacing, the advantages of contraception, and the opportunity cost of high fertility, particularly for women (Jejeebhoy 1995). Literacy and numeracy may enhance women’s abilities to navigate health bureaucracies (LeVine et al. 2012), access family planning services, and successfully use contraception.

Despite theory, and some empirical evidence (Smith-Greenaway 2013), around the important role that literacy and numeracy play in improving demographic outcomes, little research has explored what happens to these skills after girls leave school (for exceptions, see Gorman and Pollitt 1997; Soler-Hampejsek et al. 2018). In low- and middle-income countries (LMICs), given increasing school enrollment and widespread early marriage and childbearing, many adolescent girls transition rapidly from school to motherhood (Lloyd 2005). Yet, research on the effects of adolescent childbearing on education in LMICs has been limited and has focused narrowly on grade attainment.

We hypothesize that adolescent childbearing in the years after school leaving contributes to the deterioration of already weak academic skills. If true, the assumption that skills remain constant after leaving school may lead to an overestimate of the potential effects of education on economic productivity, fertility, and other demographic outcomes. For example, if literacy affects fertility through women’s access to the labor market—a commonly hypothesized pathway (Psaki et al. 2019)—rapid loss of literacy skills after leaving school may mean that adolescent girls no longer have the skills needed to join the labor market after they begin childbearing. We test our hypothesis using data from three longitudinal studies in low-income settings that followed adolescent girls during the transition from school to motherhood: the Malawi Schooling and Adolescent Study (MSAS) (2007–2013); the Bangladeshi Association for Life Skills, Income and Knowledge for Adolescents (BALIKA) study (2013–2015); and the Adolescent Girls Empowerment Program (AGEP) in Zambia (2013–2017). We compare results across these settings in part because of data availability. However, despite important social and economic differences between these settings, all three countries are facing the challenges of low-quality schools, amid rapid expansions in access to school, and high levels of adolescent childbearing. To our knowledge, this is the first study to examine the effect of adolescent childbearing on academic skills in LMICs.

## Background

Much research has documented the association between grade attainment and reproductive behavior among women in low-income countries (Bledsoe et al. 1999; Cochrane 1979): those with more years of schooling are likely to marry and give birth later and to have fewer and healthier children (Basu 2002; Cleland 2010; Gakidou et al. 2010; Glewwe 1999). This relationship may operate in either direction: for example, more schooling may contribute to delayed childbearing (Breierova and Duflo 2003; Osili and Long 2008; Smith-Greenaway 2013), and adolescent childbearing may disrupt schooling (Geronimus and Korenman 1992; Kane et al. 2013; Stange 2011). Given the many shared determinants of schooling and reproductive behavior, researchers continue to debate the extent to which these relationships are causal (Psaki et al. 2019).

Studies in low-income countries have largely focused on this relationship in one direction: the effect of education on reproductive behavior. The research that has been conducted on the effects of childbearing on education—both in low-income and high-income settings—has focused narrowly on grade attainment and has overlooked the potential effects of childbearing on academic skills.

### Mechanisms Linking Childbearing and Skills

Research and policies in low-income settings are often designed based on the assumption that academic skills remain constant after school leaving (LeVine et al. 2012), despite evidence that skills may improve (Gorman and Pollitt 1997; Hartley and Swanson 1986; Wagner et al. 1989) or deteriorate (Abadzi 2003; Durgunoğlu et al. 2003), depending on whether they are reinforced through regular use (Wagner 1994).

Several studies in LMICs have examined the trajectories of academic skill development and loss. Using a longitudinal sample from Ladino communities in Guatemala, in which respondents were first interviewed as children and followed up 20 years later, Gorman and Pollitt (1997) investigated skill loss after leaving school. They found a strong positive relationship between early grade attainment and literacy skills. In contrast to more recent data from low-income settings (World Bank 2018), all children in the Guatemala sample were literate once they had completed three years of schooling. Study respondents’ skills continued to improve after leaving school, especially among those with the lowest levels of grade attainment. More recently, research using data from the MSAS found evidence of gender differences in deterioration in literacy skills: adolescent girls had significantly lower levels of literacy in the several years after school leaving than their male peers, even after skill level at school leaving and grade attainment were controlled for. The authors suggested that students who leave school earlier, particularly females, may not have the same opportunities to apply and reinforce skills as those who leave school later (Soler-Hampejsek et al. 2018). The several years after leaving school may be a critical period for securing or strengthening academic skills for adolescent girls, who often experience the disruptions of marriage and childbearing soon after leaving school, and whose social roles may permanently change once those transitions begin (Lloyd 2005; Santhya and Jejeebhoy 2003).

Some evidence supports the idea of a threshold level of skill acquisition that protects against subsequent deterioration. Chall (1996) proposed a widely used model of language acquisition as a multistage process, from basic letter and word recognition to comprehension of single and multiple viewpoints in complex texts. This model describes a critical transition—from “learning to read” to “reading to learn”—that lays the foundation for continuous learning after leaving school or other formal learning environments. In the U.S. literature, this transition is sometimes termed the *fourth grade slump*, referring to the grade at which struggling readers often face challenges in developing more advanced comprehension skills. Others have argued that assuming a similar model of language acquisition in low-income countries, four to six years of schooling form an essential foundation to build the literacy skills necessary for lifelong learning (Wagner 1994). Therefore, even among those who leave school with basic literacy and numeracy skills, if they are unable to apply those skills because of changing roles (e.g., responsibility for childcare and housework) for a period of months or years, they may lose the skills permanently unless circumstances are altered.

Findings from the limited literature on skill loss and gain provide support for the relevance of this theory to low-income settings. In explaining skill improvements after leaving school among those with low levels of attainment, Gorman and Pollitt (1997) hypothesized that children remain in school until they gain a certain level of skills (e.g., basic literacy) in order to apply and strengthen those skills after leaving school. In the Malawi study, the authors highlighted the consistent relationship between grade attainment and skill loss, arguing that those with higher levels of grade attainment were more able to apply and strengthen their skills after leaving school (Soler-Hampejsek et al. 2018). If true, this may reflect not only stronger skills at school leaving—perhaps because learners made the critical transition to reading to learn—but also a higher likelihood of accessing employment opportunities that require the application of those skills.

Although research has shown a link between grade attainment and academic skills, albeit not as strong as expected (Psaki et al. 2019; Smith-Greenaway 2013), less evidence exists on the effects of events after school leaving on literacy and numeracy. For adolescent girls, numerous life events may occur shortly after school leaving and affect their ability to apply and strengthen the skills gained in school. We know little about the potential effects that becoming a parent—an event that often occurs earlier for women than men (Lloyd 2005) and is likely to affect the regular use of skills—has on the retention of academic skills. The question of whether adolescent childbearing affects retention of academic skills after leaving school is particularly urgent in LMICs (like those included in our study) where despite recent progress in school enrollment, attainment levels remain low (Psaki et al. 2017), school quality is poor (Kendall 2007; World Bank and UNICEF 2009), and skill levels are low overall (Chimombo et al. 2005; Pritchett 2013; Smith-Greenaway 2013; World Bank 2013, 2018).

### Effects of Adolescent Childbearing on Grade Attainment

Numerous studies, especially in high-income settings, have sought to examine the effect of adolescent childbearing on grade attainment; this research offers important lessons. Estimating the effects of childbearing on education presents a challenge given the shared underlying factors, such as socioeconomic status, that likely determine both the timing of childbearing and performance in school. Adolescent childbearing is not a random event but is instead selective by important characteristics that also affect school performance, including literacy and numeracy (Geronimus and Korenman 1992; Kane et al. 2013; Stange 2011). As a result, naïve estimates of these relationships are likely to overstate the effects of childbearing on skills. The key challenge, therefore, is to construct an appropriate counterfactual: what would have happened in terms of grade attainment and learning if a pregnancy (or birth) had not occurred (Hotz et al. 2005)?

Research on the effects of adolescent childbearing on grade attainment, largely conducted in high-income countries, has sought to address this estimation challenge by exploiting natural experiments, such as miscarriages or twins with different ages at onset of childbearing (for reviews of common approaches, see Hotz et al. 2005; Kane et al. 2013). Most studies have found statistical evidence of endogeneity in this relationship, resulting in attenuated or null effects in models addressing this issue (Ashcraft and Lang 2006; Fletcher and Wolfe 2009; Geronimus and Korenman 1992; Hotz et al. 2005; Kane et al. 2013; Webbink et al. 2011). Recent analyses using data from the National Longitudinal Study of Adolescent to Adult Health in the United States found a negative effect (0.7 years of schooling lost) of teen childbearing on educational attainment, albeit smaller than estimates from models that fail to control for endogeneity (1.0 years of schooling lost) (Kane et al. 2013). Using the National Longitudinal Survey of Youth and miscarriages as a natural experiment, Hotz et al. (2005) found no significant effect of adolescent childbearing on the probability of obtaining a high school diploma, but they found a positive effect on annual earnings.^1^ Similarly, using an Australian sample of twins, Webbink et al. (2011) found no effect of adolescent childbearing on grade attainment. Diaz and Fiel (2016) investigated heterogeneity in the effects of teen pregnancy on education and earnings and found that negative effects on earnings are most pronounced for those who are least likely to experience a teen pregnancy. More broadly, the authors noted that the effects of teen pregnancy (and childbearing) are likely to vary by the pre-pregnancy characteristics of adolescents, and this heterogeneity should be taken into account (Diaz and Fiel 2016).

Studies in LMICs have also found evidence of the selectivity of adolescent childbearing (Azevedo et al. 2012; Grant and Hallman 2008; Timaeus and Moultrie 2015). For example, research in South Africa found that adolescent girls who are struggling in school are more likely to become pregnant several years later and then drop out than their peers who perform well (Grant and Hallman 2008; Timaeus and Moultrie 2015). However, few studies in these settings have effectively addressed this selectivity in order to estimate the causal relationship between adolescent childbearing and grade attainment or other aspects of education. One exception, a study by Azevedo and colleagues, used the Mexican *Encuesta Nacional de la Dinamica Demografica* (ENADID) data set collected in 2006, which includes detailed birth, miscarriage, and abortion histories. The authors used miscarriage as a natural experiment to address endogeneity after demonstrating that women who became pregnant as adolescents in this sample are selective. Although naïve estimates found a negative effect of adolescent childbearing on educational attainment, after addressing endogeneity, the authors found a statistically significant positive effect: those who gave birth during adolescence completed 0.34 more years of schooling, on average, compared with those who became pregnant but had a miscarriage during adolescence. They also found, however, that those who gave birth during adolescence received significantly more social assistance than the comparison group (Azevedo et al. 2012).

Several studies in South Africa have also examined this question using longitudinal data, although they were unable to address unobserved determinants of childbearing and schooling (Ardington et al. 2015; Ranchhod et al. 2011). Using census data from South Africa, one study found no evidence of selectivity into teenage childbearing on observed characteristics, as well as a negative effect of childbearing on the probability of being enrolled in school at every age (Ardington et al. 2015). In contrast, using data from the Cape Area Panel Study (CAPS), Ranchhod et al. (2011) found evidence of selectivity in adolescent childbearing; they also found that despite a negative effect on high school graduation rates at age 20, this difference had disappeared by age 22, indicating a catch-up effect.

Overall, existing literature on the effects of adolescent childbearing on grade attainment shows consistent evidence of selectivity: those who give birth as adolescents differ from those who delay childbearing in important ways that may affect literacy and numeracy. However, rigorous research on this question has been concentrated in high-income settings, where the determinants of adolescent childbearing and learning likely differ from those in low-income settings. For example, compared with LMICs, most high-income countries have more widespread access to quality sexual and reproductive health services and education systems, higher levels of educational attainment and literacy, and more widely available opportunities to apply academic skills (e.g., through low-cost reading materials).

Our study builds on existing literature in three important ways. First, we examine the effects of adolescent childbearing on academic skills—a question that, to our knowledge, has not yet been investigated in the scholarly literature. Second, we use statistical methods designed to address the endogeneity in the relationship between childbearing and academic skills. Third, we conduct these analyses using data from three LMICs in two regions—Bangladesh, Zambia, and Malawi—where the determinants of adolescent childbearing and academic skills may also vary.

### Study Context

Table 1 provides nationally representative overviews of key economic, fertility, and education indicators for each country included in our study. None of the samples are nationally representative, but Table 1 provides context for our comparative analyses.

**Table 1:** Population, economy, fertility, and education characteristics of countries where each study occurred

	Bangladesh	Malawi	Zambia
Population and Economy	2016	2016	2016
Annual Population Growth (%)	1.1	2.9	3.0
GNI per Capita, Atlas Method (current US$)	1,330	320	1,300
Fertility	2014	2015–2016	2013–2014
Fertility Rate, Total (births per woman)	2.3	4.4	5.3
Adolescents (aged 15–19) Who Have Begun Childbearing (%)	31	29	29
Education: Primary Enrollment Rate (% gross)	2015	2015	2013
Female	125	147	104
Male	116	144	103
Education: Survival to Last Grade of Primary	2009	2013	2012
Female	71	55	57
Male	62	54	54
Education: Literacy Rate (%), 15- to 24-Year-Olds (2015)	2016	2015	2010
Female	94	73	87
Male	91	73	91

*Notes:* GNI per capita (formerly GNP per capita) is the gross national income, converted to U.S. dollars using the World Bank Atlas method, divided by the midyear population. *Literacy rate* is defined by the Institute for Statistics (UIS) as the percentage of people who can both read and write with understanding a short simple statement on their everyday life. *Primary gross enrollment ratio* is defined as the number of students enrolled in primary school, as a percentage of the official primary school–aged population. Survival to last grade of primary refers to the percentage of students who reach the last grade of primary school, among those who enrolled in the first grade of primary school, regardless of repetition.

*Sources:* Population and Economy data are from the World Bank’s World Development Indicators Database; fertility data are from the most recent Demographic and Health Surveys in each country; education data are from the UNESCO UIS, and dates are specified for each country and indicator in the table.

Given the challenges in finding longitudinal data that follow adolescent girls as they transition from school to childbearing, as well as data on changing skill levels, our choice to include these three countries partly reflects data availability. However, all three countries are also facing the challenges of low-quality schools, rapid expansions in access to school, and high levels of adolescent childbearing. Important differences among these countries exist as well. Although Malawi and Zambia share a border, they differ in terms of economic and educational development. In both Malawi and Zambia, adolescent childbearing often occurs outside marriage, whereas childbearing nearly always occurs in the context of marriage in Bangladesh. The comparison between these settings has the potential to shed additional light on the relationship of interest.

#### Economic Context

Bangladesh is a LMIC that has experienced sustained economic growth in recent decades and has reduced poverty levels (World Bank Group 2016). Low-skill, low-wage manufacturing jobs have driven growth, including those in the garment sector, where 90 % of employees are women. Malawi and Zambia are low-income countries that share a border in southern Africa. Malawi has experienced economic growth over the last decade with support from development partners. However, poverty continues to affect many Malawians, especially in rural areas because of the lack of opportunities outside the agricultural sector, which contributes 30 % of GDP but is subject to environmental shocks. Following a recent economic downturn in Zambia due to falling global copper prices, climate events affecting agriculture, and political uncertainty leading up to the 2016 elections, the economy of Zambia is rebounding (World Bank 2018). Growing youth populations in each country—accounting for 29 % of the population in Bangladesh and 45 % in both Malawi and Zambia in 2015—underline the urgency of ensuring that education provides the skills needed to contribute to future economic development in each country.

#### Education Context

Bangladesh operates on a 5-5-2-4 education system: 5 years of primary school, 5 years of secondary school, 2 years of higher secondary school, and 4 years of university. The official age of school entry is 6, meaning that students who enter on time and do not skip or repeat any years would complete primary school at age 10. Malawi operates on an 8-4-4 education system: 8 years of primary school, 4 years of secondary school, and 4 years of university. The official age of school entry is 6, with an expected age of 14 at the completion of primary school. Zambia operates on a 7-5-4 system, with an official age of school entry of 7, and expected primary school completion by age 14 (UNESCO Institute for Statistics (UIS) 2017).

In all three countries, alongside expanding access to school, the quality of schooling has remained poor overall and in some cases seems to have deteriorated as more students have enrolled. Following the adoption of free primary education in Malawi in 1994, enrollment increased dramatically, especially for girls and those living in the poorest households (World Bank 2018). However, school inputs have not kept up with increasing enrollment: between 2008 and 2015, as gross enrollment increased from 131 % to 145 %, the average number of students per class increased from 85 to 126 (World Bank 2018). Similar to Malawi, Zambia experienced a dramatic increase in enrollment following the elimination of primary school fees in 2002: secondary school enrollment increased by nearly 75 percentage points between 2000 and 2010, a faster rate than that experienced by any country in history. Despite increased investments in education by the government, the quality of schooling remains poor or has deteriorated amid rapid increases in enrollment. Of 14 countries, Malawi and Zambia had the highest proportion of students scored as “not competent” in both reading and mathematics based on the 2007 Southern and Eastern Africa Consortium for Monitoring Education Quality (SACMEQ 2007). By 2012, nearly 90 % of students in both countries were unable to read a single word by the end of grade 2 (World Bank 2018). Similarly, despite expanding access to education in Bangladesh, a National Student Assessment (NSA) conducted by the government in 2011 found low competency levels among grade 8 students: 44 % met standards in Bangla, 44 % met standards in English, and 35 % met standards in mathematics (World Bank 2013).

## Methods

### Data

#### Bangladeshi Association for Life Skills, Income and Knowledge for Adolescents (BALIKA) (2013–2015)

Designed as a four-arm cluster randomized controlled trial (RCT) of three interventions to delay marriage, the BALIKA study included 11,609 girls aged 12–19 at baseline living in three districts within the Khulna division of southern Bangladesh (Khulna, Narail, and Satkhira). Relative to the rest of the country, Khulna division has a lower total fertility rate but also a lower median age at first birth among adult women. The proportions of adolescents who have begun childbearing are comparable in Khulna and nationally. Khulna also has slightly better education indicators than the rest of the country, with an average of 4.3 years of education completed, compared with 3.8 nationally (NIPORT 2016).

In 2013, 96 villages and clusters within villages were selected for participation. Based on a household listing, approximately 120 girls from each cluster were randomly selected to participate; each cluster was randomly assigned to one of four study arms, resulting in 24 clusters per arm. Baseline data collection was conducted starting in August 2013 before the intervention began; endline data collection began in July 2015, after approximately 18 months of program implementation. In total, 11,609 girls responded to the baseline survey; all girls in intervention clusters were invited to participate, and 9,689 enrolled in the BALIKA program. The target sample was based on an estimated probability of keeping girls unmarried of .85 in the intervention arm and .75 in the control arm, power of .80, alpha of .05, and intraclass correlation of .03 (Amin et al. 2014). Safe space groups—used in both BALIKA and the Adolescent Girls Empowerment Program (AGEP) in Zambia—are girls’ meetings stratified by age, marital/fertility status, and sometimes schooling. Weekly meetings are led by female mentors and involve training in a core curriculum, including health and life skills, financial education, nutrition, gender rights, and early marriage. In BALIKA, all girls in intervention arms were invited to participate in safe space groups and one of the following interventions: tutoring (Arm 1), livelihoods training (Arm 2), and life skills training (Arm 3). Arm 4 was a control arm (Amin et al. 2014).

#### Malawi Schooling and Adolescent Study (MSAS) (2007–2013)

MSAS was a longitudinal cohort study of adolescents in Balaka and Machinga districts, located in the Southern region. The Southern region’s total fertility rate is higher than the national average, and its median age at first birth is younger than the national median. The median years of schooling and level of literacy for women in the Southern region are comparable to the national levels (NSO and ICF 2017). MSAS included data on adolescents, teachers, and school facilities. The baseline sample consisted of 2,649 adolescents aged 14–17 in 2007. Of these, 1,764 (875 girls and 889 boys) were students attending standards 4–8 at baseline, randomly selected from rosters of 59 randomly selected primary schools. The probability of a school being included in the sample was proportional to its 2006 enrollment. The number of schools sampled in each district was based on estimates of primary school enrollment, attendance, and attrition rates; transitions to secondary school; and dropout rates. An additional sample of 885 out-of-school adolescents was drawn from communities surrounding the selected primary schools. The ratio of adolescents in standards 4–8 relative to those out of school approximates that observed in the 2004 Malawi DHS. These analyses use only the female MSAS sample because a lower proportion of males experienced fatherhood by the last round of the study (34 %) and because we wanted to ensure comparability across studies.

#### Adolescent Girls Empowerment Program (AGEP) (2013–2017)

AGEP was a multisectoral asset-building program for adolescent girls aged 10–19 with a cluster RCT design that assessed effects on health outcomes in four provinces of Zambia. Fertility and education indicators vary across the four study provinces but, at the time the study started, were generally strongest in Lusaka and weakest in North Western province (2014 Zambia DHS; Central Statistical Office et al. 2014). AGEP interventions started in 2013 after baseline data collection and lasted for two years. There were three core components: (1) weekly safe space girls’ group meetings, (2) an adolescent-friendly savings account, and (3) a health voucher to provide access to girl-friendly health services. Clusters were randomized into four arms (Hewett et al. 2017). To investigate effects during and after the intervention, follow-up surveys were conducted annually until 2017. Ten AGEP master sites (five rural, five urban) were identified in four provinces. Within each site, 16 clusters were randomly selected and assigned to study arms through public lotteries.^2^ A household listing was conducted, and an indicator was constructed to identify the most vulnerable girls. On average, almost 30 girls per cluster were interviewed at baseline, for a total of 4,661 girls, representing an 88 % response rate of the target sample. Of these, between 84 % and 90 % were tracked in Rounds 2–4. In Round 5, because of budgetary constraints, the target sample was reduced to 3,772 girls randomly drawn from girls interviewed in Round 3; an 82 % response rate was achieved (66 % of the baseline sample). The AGEP study population was representative of vulnerable adolescent girls ages 10–19 in the study areas at baseline; it was also representative of the 10–14 and 15–19 age groups, separately by urban and rural areas (Hewett et al. 2017). Descriptive and analytical findings for the current study are separated by the AGEP urban and rural samples based on the assumption that these relationships may operate differently in each setting; separate analyses by rural/urban area also allow for a more direct comparison between the rural AGEP sample and the other two studies, both of which were conducted in rural settings.

Table S1 (online appendix) summarizes information about the design and sample of each study included in our analyses.

### Dependent Variables

We examine the effects of childbearing on several academic skills: basic oral reading skills in English and a local language and basic numeracy skills (in MSAS and BALIKA only).

#### Literacy

Literacy assessments were nearly identical in the three studies. In English and a local language,^3^ respondents were asked to read aloud two simple sentences drawn from the most recent Demographic and Health Survey (DHS) conducted in each country. If they were unable to read each whole sentence, the interviewer probed and asked whether they could read any part of the sentence. Responses on each sentence were scored as 0 if they were unable to read the sentence at all, 1 if they were able to read part of the sentence, and 2 if they were able to read the whole sentence. Responses on two sentences were summed for scores ranging from 0 to 4 for each reading assessment. We then standardized scores within each sample based on the baseline values because we are more interested in relative changes in scores than absolute score values.

The same sentences were used in each round for all three studies.^4^ In AGEP (Zambia), the first sentence in the local language assessment was in Nyanja, and respondents were given a choice of Nyanja, Bemba, or Kaonde (the most common languages in the study sites) for the second sentence. In BALIKA (Bangladesh), participants were first asked whether they knew how to read or write. If they responded that they did not know how to read, they skipped the literacy assessments and were categorized as illiterate in both English and Bangla.

#### Numeracy

In MSAS and BALIKA, numeracy skills were also assessed in each round, albeit somewhat differently. In MSAS, respondents were asked to complete a short numeracy assessment, including filling in missing numbers, ordering numbers, basic addition, subtraction, multiplication, and division. They were also asked two money word problems. Questions were drawn from the Malawi Institute of Education (MIE) achievement tests for standard 3.^5^ Two slightly different versions of the assessment, with the same questions but different numbers, were administered in alternating rounds. BALIKA used a similar but more complex numeracy assessment, including 20 questions, some of which covered more advanced numeracy concepts than the MSAS assessment. To compare results between the two studies, we use a summary score of eight questions, comparable versions of which were included in both MSAS and BALIKA (see Table S5, online appendix). These questions included filling in missing numbers, ordering numbers, addition, subtraction, multiplication, division, and two simple word problems. In each study, the numeracy assessments were administered in the local language: Bangla in Bangladesh and Chichewa in Malawi. In contrast to the other studies, which included the numeracy assessment in every round, it was included only in alternate rounds of AGEP; we do not include AGEP numeracy results in this study.

### Independent Variables

We examine the effects of first birth on academic skills. In each round, respondents were asked whether they had ever given birth, and were coded as having never given birth (0) or ever given birth (1). For all studies, random-effects models also include age at baseline. In addition, in our fixed-effects models, we control for a series of time-varying independent variables that are likely to be associated with both academic skills and the timing of childbearing:

- Grade attainment, measured as the last grade attended at the time of the survey in MSAS and BALIKA, and the last grade completed at the time of the survey for AGEP. Because the length of primary school differs across the study countries, we code grade attainment as grade 5 or less, grade 6, grade 7, grade 8, and grade 9 or more.^6^
- Time since school leaving, coded as 0 every round a respondent was still attending school, 1 in the first round after she left school (0–1 years out of school), 2 in the subsequent round (1–2 years), and so on. Because BALIKA has only two rounds and its analytic sample includes girls who were attending school at baseline, respondents are coded as either still attending school at endline or having left school between baseline and endline.
- Current work status, defined as work done on a regular basis, excluding household chores, regardless of whether the work is paid.^7^
- Household wealth, measured as a sum of household items, based on the eight assets included in all three studies: mattress/cot/bed, table, TV, radio, mobile phone, bicycle, motorcycle, and electricity.

Although the primary research questions for the AGEP and BALIKA studies were focused on evaluating programs, we analyze these data as if they are drawn from observational studies, meaning that exposure to the intervention is a nuisance factor that we control for in our statistical models. We take several steps to confirm that the interventions did not affect our findings. First, in both the fixed and random-effects models using AGEP data, we adjust for exposure to the program, which is time-varying. Although we do not have data on program exposure in BALIKA, we adjust for program arm assignment, which is fixed, in our random-effects models. Given the larger sample size in the BALIKA study, we also rerun the BALIKA models in the control arm only to confirm that the results are consistent.

### Analytic Strategy

We begin our analyses by providing descriptive information about the full sample of females included in each study to motivate the key research question. We describe patterns of basic literacy skills by grade attainment and time since school leaving.

Our regression analyses use only the sample of students from each study who were in school at baseline because of the lack of data on skill levels at school leaving for those who left school prior to baseline. The analytic sample is also limited to those who had not yet experienced a first birth at baseline. This sample is selective, excluding those at highest risk of skill loss and adolescent pregnancy, which is an unavoidable consequence of the study designs and highlights the need for more studies that follow children from school entry through adulthood. The nature of these samples may lead to more conservative estimates of the effects of childbearing on academic skills. We report descriptive information on education and demographic variables for the analytic samples.

We estimate the effect of the occurrence of the first birth on skill level controlling for key covariates using the following model:

$$ {S}_t={\upbeta}_0+{\upbeta}_1{R}_t+{\upbeta}_2{G}_t+{\upbeta}_3{L}_t+{\upbeta}_4{G}_t{R}_t+{\upbeta}_5{\mathbf{X}}^O+{\upbeta}_6I+u+{v}_t, $$

where *S*_*t*_ represents skill level at time *t*; *R*_*t*_ represents whether the respondent had a first birth by time *t*; *G*_*t*_ represents grade attainment by time *t*; *L*_*t*_ represents years since school leaving at time *t*; **X**^*O*^ represents additional observed controls for the dependent variable of interest (e.g., household wealth, employment status); *I* represents intervention arm for BALIKA (measured as assignment) and program exposure for AGEP (measured as accumulated number of meetings attended by time *t*); *u* represents unobserved determinants of both the outcome and key independent variables (e.g., innate ability, interest in schooling); and *v*_*t*_ is a random disturbance term. We include a hypothesized interaction between grade attainment (*G*_*t*_) and first birth (*R*_*t*_) to assess whether the effect of a birth on skill level varies by level of grade attainment, given previous evidence and theory supporting this relationship (Chall 1996; Gorman and Pollitt 1997; Soler-Hampejsek et al. 2018). Time (*t*) represents years, where *t* = 0 is the first round of data collection in each study, for those who were unmarried and had never had a birth at baseline. Possible values of *t* reflect years of follow-up.

Longitudinal data are useful in establishing the temporality of events, although observed associations may reflect unobserved determinants of both skill levels and key independent variables rather than true effects. Because we cannot differentiate *u* from *v*_*t*_ in the empirical estimates of the relations described earlier, we use fixed-effects models to obtain unbiased estimates of the parameters of interest by adjusting for time-invariant, unobserved characteristics of the respondents or their environments (e.g., innate ability) that might otherwise lead to biased estimates. We compare the fixed-effects and random-effects results using Hausman tests to confirm that the fixed-effects models are the most appropriate choice for estimating the relationships of interest (Allison 2009).

### Missing Data

Levels of missing data were low in the analytical samples for MSAS (<1 %) and AGEP (4 %) but varied by selected baseline characteristics. Because both MSAS and AGEP included more than two rounds of data collection, we consider those lacking complete data in at least two rounds as missing given that those observations did not contribute to models. In MSAS, those excluded from analyses because of missing data had lower Chichewa reading scores at baseline than those with complete data; all other baseline characteristics were comparable. In AGEP, those excluded from analyses because of missing data had lower English reading scores and fewer household items at baseline; all other characteristics were comparable. In BALIKA, levels of missing data were higher (11 %) and differed by multiple baseline characteristics. As a robustness check, we reran all models for each study using complete cases only and inverse probability weighting to account for the selectivity of missing data. The results were consistent for AGEP and MSAS, so we report the unweighted models. Although the results for BALIKA were consistent overall, we report the weighted models given the higher levels of missing data.

As an additional robustness check, we reran the models for AGEP and MSAS using fully balanced panels, including only the observations with complete data in every round of follow-up. This resulted in reduced samples: 73 % of the analytical sample for MSAS, and 63 % of the rural and 71 % of the urban analytical samples for AGEP. The results were consistent with those for the full analytical samples (available on request), which indicates that among those in the analytical sample, data were missing at random. We therefore maintain the full analytical samples in our final tables.

## Results

### Descriptive Results

**Fig. 1:**
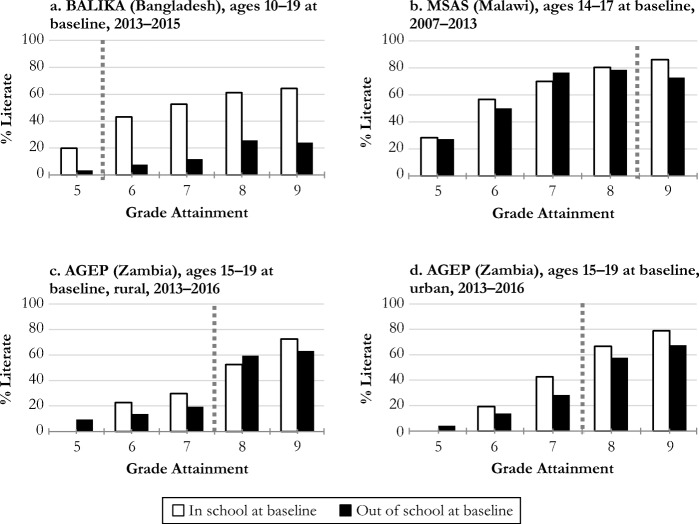
English oral reading skills by baseline school enrollment status. The figure presents the measurement of literacy skills that occurred most closely to the time of school leaving, stratified by school enrollment status at the first round of data collection. That is, for girls who were out of school at the first round of data collection (baseline), the figure reflects literacy at that point; for girls who were in school at the first round of data collection and dropped out during follow-up, the figure reflects literacy assessment conducted in the first round after they left school. The dotted line represents the transition from primary to secondary school. Only six girls in the AGEP urban sample and 12 girls in the AGEP rural sample were enrolled in school at baseline and then dropped out immediately after completing grade 5.

Figure 1 shows the relationship between grade attainment, literacy (English), and baseline school enrollment status in each study. In each panel, the white bars represent the sample of girls who were enrolled in school at the first round of data collection (baseline), and the black bars represent those who had already dropped out of school by baseline. We use school enrollment status at baseline as a proxy for time since leaving school. Girls who were out of school at baseline had been out of school for an average of approximately 2 years in MSAS (2.0) and the AGEP rural sample (2.2), and closer to an average of 3 years (2.8) in the AGEP urban sample. We do not have this information for the BALIKA out-of-school sample. To begin exploring what happens to skills after leaving school, we compare literacy levels, by grade attainment, between these two groups. The bars show the percentage literate within each group by level of grade attainment.

Overall, Fig. 1 demonstrates two important patterns in our data: (1) literacy levels are low overall and increase with grade attainment, particularly in the transition to secondary school; and (2) literacy levels are lower among those who have been out of school longer, even at comparable levels of grade attainment. This difference may reflect multiple factors: skills in this group may have been weaker at school leaving, deteriorated after school leaving (Soler-Hampejsek et al. 2018), or both. It is also possible that those out of school longer were weaker students, even before leaving school, than those who were still attending school at baseline. We explore these relationships in more detail for the in-school samples in our analytical models.

Table 2 shows the baseline characteristics of the full samples in each study as well as the final analytical samples. Although BALIKA included a wider age range (12–19) than MSAS (14–17) or AGEP (15–19),^8^ the proportion out of school at baseline was comparable across the samples. By design, the AGEP sample included only unmarried girls at baseline. However, the proportion of respondents who had ever given birth at baseline was similar across the three studies, reflecting the different contexts of childbearing across settings. Despite the young ages and selectivity of the samples, many respondents in the analytical samples in each study had a first birth during follow-up: 474 in BALIKA, 639 in MSAS, 193 in the AGEP urban sample, and 245 in the AGEP rural sample (not shown).

**Table 2:** Baseline characteristics of full samples, and final analytical samples, by study

	BALIKA (Bangladesh)	MSAS (Malawi)	AGEP (Zambia)	
			Rural	Urban
Total Study Sample	11,609	1,337	1,062	1,193
% Ever gave birth	7	10	12	10
% Out of school	22	35	27	35
% Not eligible (ever gave birth and/or out of school)	23	36	30	37
% Eligible, but missing data	11	<1	4	4
Final Analytical Sample	7,698	856	697	706
% of full female sample	66	64	66	59
Age range	12–19	14–17	15–19	15–19
Highest grade attended (%)				
Grade 5 or less	16	21	10	9
Grade 6	16	23	12	18
Grade 7	17	27	20	22
Grade 8	14	29	30	31
Grade 9 or more	37	–	29	20
% Able to read in English	53	73	61	64
% Able to read in local language	92	93	51	38
Mean numeracy score (range: 0–8)	6.7	6.1	4.8	4.7
% Currently working	7	23	29	16
Mean number of household assets	4.3	2.6	4.3	4.6

*Notes:* The analytical samples include only those study participants who were in school at baseline because of a lack of data on skill levels at school leaving for those who left school prior to baseline. The analytical sample is also limited to those who had not yet experienced a first birth at baseline. There are further restrictions to the analytical sample by study: we exclude data on 1,312 males collected in MSAS because of the lower proportion of males experiencing a first reproductive event by Round 6 (39 % were ever married, and 34 % ever had a biological child) and because we wanted to ensure comparability across studies. We also exclude data on the sample of 10- to 14-year-old girls at baseline in AGEP because childbearing data were not collected until age 15. For each study, a small number of additional observations are excluded because of missing data. Data on the following eight household assets were collected across all three studies: mattress/cot/bed, table, TV, radio, mobile phone, bicycle, motorcycle, and electricity. This table includes the mean number of household assets in the analytical sample, of a total of these eight, by study.

Our analytical samples exclude all girls who were out of school or had already given birth at baseline. We also exclude a small number of girls who were missing data on key variables. The resulting analytical samples range from 59 % to 66 % of the full female samples, but they reflect between 90 % and 98 % of the in-school (at baseline) female samples. As noted, these samples are selective: they do not capture the most vulnerable girls who dropped out of school before baseline or who never attended school. In the case of MSAS, where girls were recruited if attending the last four years of primary school, the sample also excludes the highest performing students in this age group, who were already enrolled in secondary school.

### Analytical Results

#### Literacy

Table 3 presents the results from fixed-effects linear regression models estimating the effect of first birth, grade attainment, and a series of covariates on English literacy score in each study. In all three rural samples (BALIKA, MSAS, and the rural AGEP sample), we find evidence of significant negative effects of time since school leaving on literacy scores; in the urban AGEP sample, this effect is significant but positive. We find less consistent evidence of effects of household wealth or work status on English literacy (not shown).

**Table 3:** Estimated effect of a birth on standardized English oral reading score from linear fixed-effects models

	BALIKA (Bangladesh)		MSAS (Malawi)		AGEP (Zambia)			
					Rural		Urban	
	Model 1	Model 2	Model 1	Model 2	Model 1	Model 2	Model 1	Model 2
Ever Birth	–0.06	0.05	–0.03	0.05	–0.05	–0.003	–0.07	–0.09
Grade Attainment								
<6 grades	–0.78***	–0.79***	–0.67***	–0.66***	–0.75***	–0.82***	–0.66***	–0.66***
6 grades	–0.38***	–0.37***	–0.22***	–0.17**	–0.61***	–0.60***	–0.39***	–0.38***
7 grades	–0.26***	–0.26***	–0.10***	–0.05**	–0.30***	–0.28***	–0.21***	–0.22***
8 grades	–0.11***	–0.10***	–0.04*	–0.03	–0.09**	–0.07*	–0.05^†^	–0.06*
9+ grades (ref.)	––	––	––	––	––	––	––	––
Ever Birth × Grade Attainment								
Birth × <6 grades		–0.05		–0.24*		0.38		–0.28*
Birth × 6 grades		–0.55***		–0.30**		–0.35^†^		–0.13
Birth × 7 grades		–0.27*		–0.17**		–0.22*		0.14
Birth × 8 grades		–0.24*		0.00		–0.11		0.06
Birth × 9+ grades (ref.)		––		––		––		––
Hausman Test Results (fixed-effects vs. random-effects models), *p* Value		.0001		.0001		.0001		.0001
Number of Observations	15,276		4,776		3,066		3,157	
Number of Groups (subjects)	7,638		856		697		706	

*Notes:* Raw oral reading score ranges from 0 to 4 (for each sentence, 0 = cannot read at all, 1 = can read partial sentence, 2 = can read full sentence; scores on each sentence are summed). Regression models use standardized reading score values based on the baseline distribution of scores in each study. All models adjust for geographic clustering. BALIKA models use inverse probability weighting to account for loss to follow-up at endline. Because BALIKA included only two rounds of data collection, time since school leaving is dichotomous, coded as either in school or out of school at endline. Models control for time since school leaving, current work status, and household wealth. AGEP models also control for time-varying exposure to the intervention, but this information was not available for BALIKA.

^†^*p* < .10; *p < .05; **p < .01; ***p < .001

**Fig. 2:**
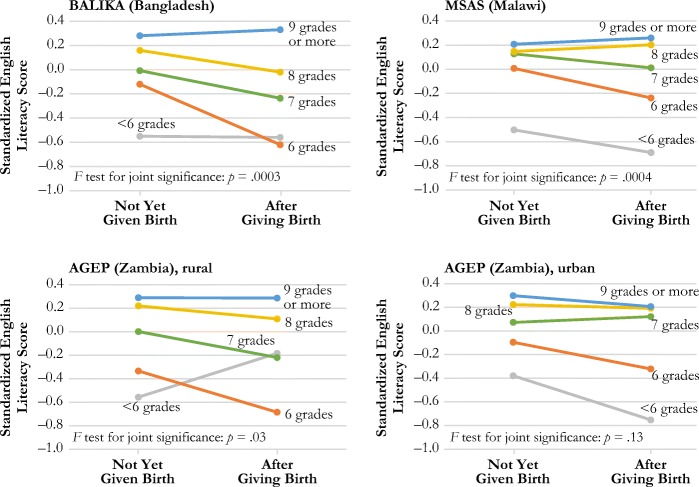
The effect of birth status on standardized English literacy by level of grade attainment

Our final models include evidence of significant interactions between birth status and grade attainment in all four samples. Using the model results shown in Table 3, Fig. 2 shows how the effects of birth status on English literacy vary by level of grade attainment in each study. Results from *F* tests show that these interaction effects are jointly significant in all three rural samples, but not in the urban Zambia sample, despite showing a similar pattern. In MSAS and BALIKA, among those with low levels of grade attainment (less than grade 6), giving birth has a significant negative effect on English literacy. However, for students who reach the last year of primary school or transition to secondary school, this effect reverses direction, and those who have ever had a birth have slightly higher English literacy scores than those who have not had a birth.

In AGEP, the patterns differ slightly between the urban and rural samples but are overall consistent with those seen in the MSAS and BALIKA samples. In the rural sample, among those with less than grade 6 attainment, those who have ever had a birth have higher English literacy scores than those who have never had a birth, but this difference is not significant.^9^ This relationship reverses for grade 6 and grade 7 attainment, when having a birth has a negative effect on English literacy; by secondary attainment (grade 8 and above) there is no difference between groups. In the urban sample, having a birth has a negative effect on English literacy for those with low attainment (less than grade 6), but there is no difference at higher levels of grade attainment.

**Fig. 3:**
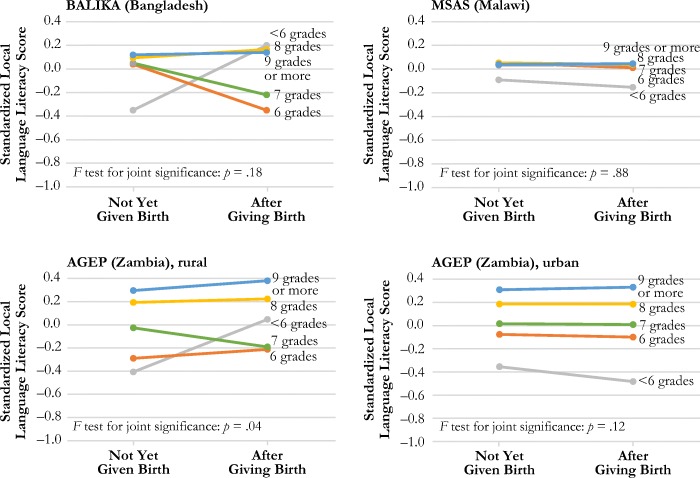
The effect of birth status on standardized local language literacy score by level of grade attainment

Table 4 presents the results from fixed-effects linear regression models estimating the effect of first birth, grade attainment, and a series of covariates on local language literacy score in each sample. Using those results, Fig. 3 shows the estimated effects of childbearing on local language literacy by level of grade attainment in each study. In MSAS, BALIKA, and the AGEP urban sample, the main effects of childbearing on local language literacy are not significant, nor are the joint interaction effects of grade attainment and childbearing on local language literacy. In the AGEP rural sample, the results show a significant interaction between grade attainment and childbearing, indicating that childbearing has a negative effect on local language literacy for those with lower levels of grade attainment and no effect—or perhaps even a positive effect—for those with higher levels of grade attainment.^10^,^11^

**Table 4:** Estimated effect of birth on standardized local language oral reading score from linear fixed-effects models

	BALIKA (Bangladesh)		MSAS (Malawi)		AGEP (Zambia)			
					Rural		Urban	
	Model 1	Model 2	Model 1	Model 2	Model 1	Model 2	Model 1	Model 2
Ever Birth	–0.03	0.02	–0.01	0.01	0.05	0.09^†^	0.01	0.02
Grade Attainment								
<6 grades	–0.45***	–0.46***	–0.13^†^	–0.12^†^	–0.65***	–0.69***	–0.65***	–0.65***
6 grades	–0.08*	–0.07*	0.00	0.02	–0.55***	–0.58***	–0.38***	–0.38***
7 grades	–0.07*	–0.06*	–0.01	0.00	–0.34***	–0.31***	–0.29***	–0.28***
8 grades	–0.02	–0.02	0.01	0.02	–0.12**	–0.11**	–0.12**	–0.12**
9+ grades (ref.)	––	––	––	––	––	––	––	––
Ever Birth × Grade Attainment								
Birth × <6 grades		0.12		–0.07		0.37†		–0.15*
Birth × 6 grades		–0.42^†^		–0.05		–0.01		–0.05
Birth × 7 grades		–0.30		0.00		–0.25*		–0.03
Birth × 8 grades		0.05		–0.02		–0.05		–0.02
Birth × 9+ grades (ref.)		––		––		––		––
Hausman Test Results (fixed-effects vs. random-effects models), *p* Value		.0001		.0001		.0001		.0001
Number of Observations	15,276		4,776		3,066		3,157	
Number of Groups (subjects)	7,638		856		697		706	

*Notes:* Raw oral reading score ranges from 0 to 4 (for each sentence, 0 = cannot read at all, 1 = can read partial sentence, 2 = can read full sentence; scores on each sentence are summed). Regression models use standardized reading score values based on the baseline distribution of scores in each study. All models adjust for geographic clustering. BALIKA models use inverse probability weighting to account for loss to follow-up at endline. Because BALIKA included only two rounds of data collection, time since school leaving is dichotomous, coded as either in school or out of school at endline. Models control for time since school leaving, current work status, and household wealth. AGEP models also control for time-varying exposure to the intervention, but this information was not available for BALIKA.

^†^*p* < .10; *p < .05; **p < .01; ***p < .001

#### Numeracy

**Fig. 4:**
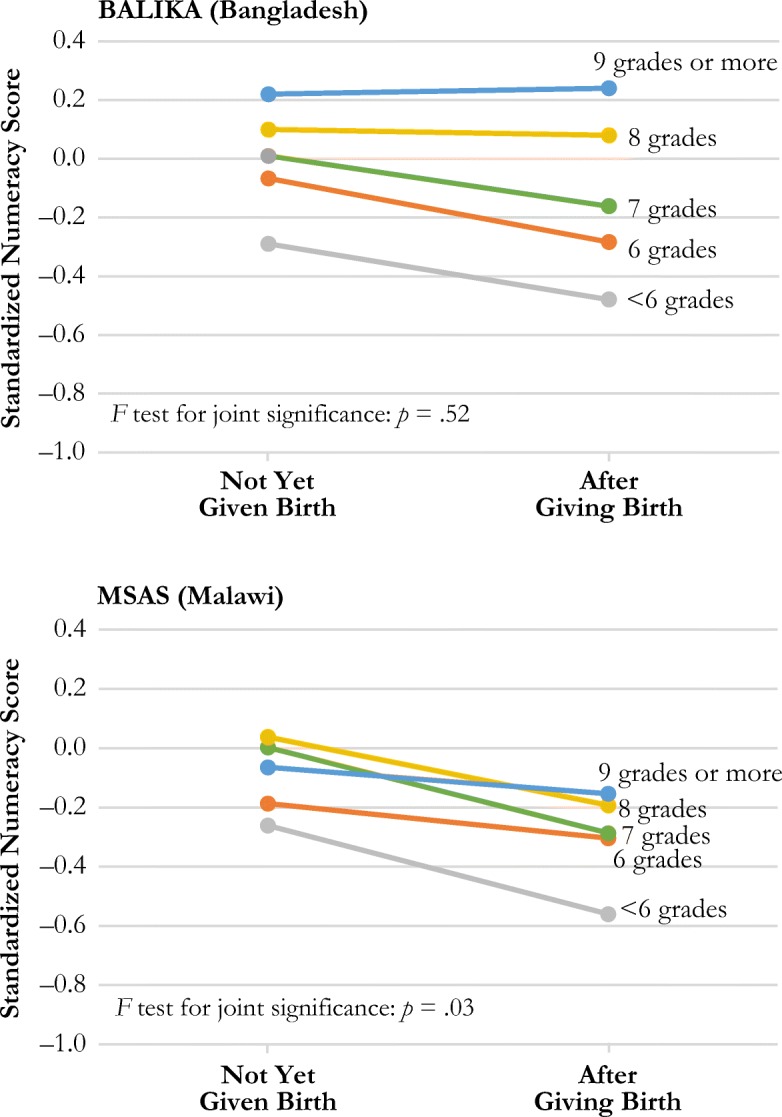
The effect of birth status on standardized numeracy score by level of grade attainment

Table 5 shows the results from fixed-effects linear regression models estimating the effects of first birth, grade attainment, and a series of covariates on numeracy score in the MSAS and BALIKA samples. Using the results shown in Table 5, Fig. 4 displays the differing effects of birth on numeracy skills by grade attainment level in each study. Although the joint interaction is statistically significant only for MSAS, the results for both studies are consistent with the English literacy findings. At low levels of grade attainment, childbearing has a significant negative effect on numeracy skills; this effect is null for those with higher levels of grade attainment.

**Table 5:** Estimated effect of birth on standardized numeracy score from linear fixed-effects models

	BALIKA (Bangladesh)		MSAS (Malawi)	
	Model 1	Model 2	Model 1	Model 2
Ever Birth	–0.02	0.02	–0.18	–0.09^†^
Grade Attainment				
<6 grades	–0.90***	–0.47***	–0.18^†^	–0.17
6 grades	–0.49***	–0.26***	–0.10^†^	–0.10
7 grades	–0.38***	–0.18***	0.05	0.08
8 grades	–0.23***	–0.11***	0.09**	0.12**
9+ grades (ref.)		––		––
Ever Birth × Grade Attainment				
Birth × <6 grades		–0.21		–0.21
Birth × 6 grades		–0.24		–0.24
Birth × 7 grades		–0.20		–0.20
Birth × 8 grades		–0.04		–0.04
Birth × 9+ grades (ref.)		––		––
Hausman Test Results (fixed-effects vs. random-effects models), *p* Value		.0001		.0001
Number of Observations	15,276		4,789	
Number of Groups (subjects)	7,638		856	

*Notes:* Raw numeracy score ranges from 0 to 8 (one point for each of eight questions answered correctly). Regression models use standardized numeracy score values based on the baseline distribution of scores in each study. Models adjust for geographic clustering. BALIKA models use inverse probability weighting to account for loss to follow-up at endline. Because BALIKA included only two rounds of data collection, time since school leaving is dichotomous, coded as either in school or out of school at endline. Models control for time since school leaving, current work status, and household wealth.

^†^*p* < .10; **p < .01; ***p < .001

Table 6 summarizes our findings on the effects of childbearing on academic skills by grade attainment level, skill, and study. Among those with low levels of grade attainment (grade 7 or lower), we find an overall pattern of negative effects of childbearing on English literacy and numeracy but less consistent effects on local language literacy. Among those with higher levels of grade attainment, childbearing tends to have a smaller negative effect or no effect on skills overall. Reflecting these patterns, we find consistently significant interaction effects between grade attainment and childbearing across all three studies.

**Table 6:** Summary of results by skill, grade attainment level (low or high), and study

				AGEP (Zambia)	
Grade Attainment	Skill	BALIKA (Bangladesh)	MSAS (Malawi)	Rural	Urban
High Attainment (grade 8 or higher)					
	English literacy	None*	None*	None*	None
	Local language literacy	None	None	None*	None
	Numeracy	None	None*	––	––
Low Attainment (grade 7 or lower)					
	English literacy	Negative*	Negative*	Negative*	Negative
	Local language literacy	Negative	None	Mixed*	None
	Numeracy	Negative	Negative*	––	––

*Notes:* In several cases, the <grade 6 attainment group experienced a different pattern from the rest of the sample. This summary table shows overall trends, despite these exceptions for the lowest grade attainment group.

*Statistically significant interaction between grade attainment and childbearing for each learning outcome.

For both AGEP and BALIKA, we find that the assignment or exposure to the program had only a very small, often nonsignificant, effect on skill levels. We adjust for those effects in our models. Given the very large sample, we also reran the BALIKA models in the control arm only and found that our conclusions were maintained (results not shown).

## Discussion

Over the last few decades, government policies in LMICs, particularly those supporting free compulsory schooling, have led to dramatic expansions in school enrollment, especially for girls (Behrman 2015; Grant 2015; World Bank 2013, 2018). As a result, many more girls are now enrolling in school, and remaining there during adolescence, than several decades ago (Lloyd 2009; UNESCO 2016). Recent evidence has also demonstrated that despite progress in enrollment, young people in many settings are unable to gain even basic academic skills during primary school (Smith-Greenaway 2013; World Bank 2018), and many fail to complete primary school and continue onto secondary school (Psaki et al. 2017; UNESCO 2016). Beyond the immediate benefits of education, many policy-makers and researchers expect that expanded access to schooling will translate into lower fertility across diverse contexts and populations, with mixed empirical support for this assumption (Behrman 2015; Breierova and Duflo 2003; Grant 2015; Osili and Long 2008). Evidence on the mechanisms linking education and fertility in LMICs is also inconsistent (Psaki et al. 2019). Therefore, questions remain as to the circumstances under which expanded access to schooling for girls is most likely to translate into lower levels of fertility and other demographic benefits.

We find support for our hypothesis that adolescent childbearing has a negative effect on academic skills, with important differences by level of grade attainment and skill type. Among those with low attainment (grade 7 and below), childbearing has consistently negative effects on English literacy and numeracy. Among those with higher levels of attainment (grade 8 and above), childbearing tends to have smaller effects or no effects on skills overall. The effects of childbearing on local language literacy are smaller or nonexistent. Despite the apparent protective effect of secondary school against skill loss, we also find that basic academic skills are not universal, even among those who have attended secondary school. For example, in the Zambia study, among those enrolled in the first year of secondary school at baseline, only 54 % of the rural sample and 69 % of the urban sample could read two full sentences in English (see Fig. 1). This finding runs counter to the assumption that all those who have attended some secondary school are literate.^12^

Our results suggest that attaining grade 8 or higher—reflecting secondary school in Malawi and Zambia, and upper secondary in Bangladesh—is protective against skill loss due to childbearing. This evidence of a threshold level of grade attainment that may secure academic skills—especially English literacy and numeracy—is consistent with existing theory on how literacy skills are acquired (Chall 1996; Wagner 1994). Our results may initially appear inconsistent with those of Gorman and Pollitt (1997), who found that skills continued to improve for young people in Guatemala after leaving school, and the largest improvements were for those with the lowest levels of attainment. Importantly, however, every respondent in their sample who attained at least three years of schooling was literate, perhaps in part because Spanish (the language of assessments) is commonly spoken in Ladino communities in Guatemala, where the data used for this study were collected.

In contrast, in our samples, a much smaller proportion of respondents with low levels of attainment had basic English literacy and numeracy skills. For example, in the MSAS analytical sample, only 8 % of those with grade 4 attainment had basic English literacy skills, compared with 85 % of those with grade 8 attainment (not shown). Therefore, although the pattern of skill retention after school leaving differs between our study and the work in Guatemala (Gorman and Pollitt 1997), both are consistent with a threshold model of skill acquisition and loss, where thresholds differ between settings and by skill.

The data from AGEP/Zambia also provide the opportunity to compare these relationships between urban and rural areas, where the contexts of adult literacy and opportunities for adolescent mothers differ. The English literacy results in both rural and urban Zambia are consistent with those from the other rural samples (MSAS and BALIKA): we find an overall negative effect of childbearing on English literacy among those with low attainment and less consistent effects among those with high grade attainment. Notably, however, time since school leaving in the urban Zambia sample has a significant and positive effect on English literacy, in contrast to the significant and negative effect found in the rural samples in all three studies (see Table S2, online appendix). The experiences of young people after leaving school, and specifically their opportunities to apply and strengthen skills, are likely different in the urban and rural samples. Smith-Greenaway (2017) explored links between community education context (specifically, the proportion of women who are literate) and child survival using DHS data from 30 sub-Saharan African countries. She found that the proportion of literate women in a community was even more important than mother’s literacy and that the relationship between community context and child survival did not depend on mother’s literacy level. Instead, she argued that this relationship may reflect the effects of the presence of literate women in a community on social learning and influence as well as shifting the institutional context. Our findings on the differences between rural and urban samples in Zambia provide support for the idea that community-level factors—such as access to reading materials, or expectations about mobility and social and economic engagement by mothers—may influence the effects of adolescent childbearing on literacy and numeracy. More research is needed on the role of community context in modifying this relationship.

We also find interesting differences in the effects of childbearing on English versus local language literacy. The results are particularly striking in the Malawi sample, where even grade attainment and current school enrollment status were unrelated to local language literacy. In the Zambia samples, levels of local language literacy were low at baseline, likely reflecting the fact that more than twice as many respondents spoke Bemba at home (65 %) than Nyanja (31 %), and the first sentence in the local language assessment was in Nyanja.^13^ In both Zambia samples, the effects of childbearing on local language literacy are more similar to the results for English literacy, likely reflecting the fact that these skills were more tenuous in Zambia because of a greater diversity in languages spoken at home. In contrast, the high levels of local language literacy in Bangladesh and Malawi, coupled with the lack of effects of childbearing on these skills, indicate that those skills were likely acquired outside of school and were more easily maintained through daily activities after leaving school.

The education community, including UNESCO and national governments, has increasingly emphasized the importance of instruction in learners’ mother tongue (that is, language spoken at home) for several reasons: growing evidence that it improves learning outcomes, inclusion of diverse learners and community members, and maintenance of linguistic and cultural diversity (Trudell 2016; UNESCO 2007). At the same time, English language skills are often seen as not only economically valuable (given that English is often required for entry into higher education and the formal economy) but also symbolically appealing (Casale and Posel 2011; Chowdhury and Kabir 2014). Research on the economic returns to “dominant” language skills (often English) has focused largely on the value to immigrants looking for jobs in the formal labor market in high-income countries. However, Casale and Posel (2011) found evidence of substantial economic returns to English literacy for Africans in South Africa and limited evidence of independent returns to local language literacy, although the two were closely related. In populations that are less likely to enter the formal labor market, such as those included in our study, local language literacy and numeracy skills may be more valuable than English language literacy for commerce, communication, and daily life (Trudell 2016).

Previous research has explored both biological and behavioral reasons that childbearing may contribute to deterioration of weak academic skills. Most of the research focused on the biological effects of pregnancy and childbearing on maternal cognition has been conducted in high-income countries and in lab settings. Researchers have described several plausible biological pathways contributing to deteriorating cognition during pregnancy and the postpartum period, including lack of sleep, depressed mood, and changes in hormone levels (De Groot 2006). Results have been mixed, with some studies finding negative effects of pregnancy and childbearing on cognitive function (De Groot 2006) and others finding differences only in reported cognition rather than objective performance (Casey 2000; Crawley et al. 2003). A 2007 systematic review found evidence of small but significant memory deficits among pregnant and postpartum women, particularly for tasks that placed high demands on respondents, such as free recall (retention in the absence of cues) and delayed free recall tasks (Henry and Rendell 2007). A prospective study with first-time mothers found substantial changes in brain structure during pregnancy in the region linked with social cognition, which has been found to be important for attachment. These changes were sustained for two years in new mothers. The same changes were not observed in first-time fathers, indicating a possible biological link.^14^ The authors speculated that this restructuring process may contribute to cognitive deficits for pregnant and postpartum women (Hoekzema et al. 2017).

The included studies followed respondents for only a few years after they began childbearing, meaning they likely had not completed childbearing during follow-up. Although we are able to observe negative effects of childbearing on skills in that relatively short time frame, it is possible that those effects were temporary and that skills rebounded once childbearing was complete. A rebound in skills would be most plausible if negative effects were completely due to temporary biological changes, including those due to exhaustion. Given the time frame of follow-up—years rather than months for most study respondents—we believe it is more likely that the negative effects of childbearing on skills reflect changes in social roles, which are likely maintained after the first birth. Therefore, a rebound in skills seems less likely; rather, we may expect to see further deterioration in skills with longer follow-up. Some research in the United States (Hotz et al. 2005) and South Africa (Ranchhod et al. 2011) has found that the negative effects of childbearing on grade attainment are short-lived and that women are able to catch up to their peers by adulthood. In both of these settings, adolescent mothers commonly return to school after giving birth, allowing women to catch up with their peers. In all three settings included in these analyses, it is uncommon for young women to return to school after beginning childbearing.

Adolescent pregnancy and childbearing often leads to immediate and long-term shifting in social roles (Lloyd 2005), which may be most pronounced and sustained in settings with unequal gender norms around childrearing (Santhya and Jejeebhoy 2003). Few studies have investigated the social consequences of adolescent childbearing in LMICs. One exception, a qualitative study in India, found that married adolescent girls reported increased restrictions on their mobility and social interactions after marriage. The authors argued that compared with their unmarried peers and older women, married adolescents were less likely to be exposed to new ideas, resources, and even diverse topics of conversation (Santhya and Jejeebhoy 2003). Although the experience of adolescent childbearing is almost certainly selective, once a birth occurs, motherhood and school attendance are incompatible in most LMICs (Eloundou-Enyegue 2004; Lloyd and Mensch 2008). Therefore, narrowing opportunities to apply skills and limited exposure to new information may explain negative effects of adolescent childbearing on academic skills. If so, expanding adolescent mothers’ mobility and opportunities to apply their skills may protect against skill loss.

Regardless of the mechanisms explaining this relationship, skill loss after school leaving among adolescent girls may have important repercussions for adult literacy, economic productivity, and fertility. Literature in high-income countries has explored the motherhood wage penalty, seeking to identify the factors that account for lower earnings among women with children (compared with their childless counterparts). Common explanations have focused on (1) loss of work experience, (2) lost productivity at work, (3) trade-off of higher wages to focus on mother-friendly jobs, and (4) discrimination by employers (Budig and England 2001; Correll et al. 2007). Although conducted in very different economic and social contexts, this area of research raises questions about the longer-term economic impact of motherhood in LMICs, especially when childbearing begins during adolescence. Potential effects of childbearing on loss of skills are not explicitly captured by lost productivity theories, which tend to focus more on behavioral differences (less sleep, more distraction) (Budig and England 2001). Even in settings with less formal labor markets, however, lost skills very likely translate into a wage penalty for mothers (or perhaps a more general economic penalty), which may be most pronounced for adolescent mothers who were unable to secure employment before beginning childbearing.

In terms of effects on child health, young mothers with weakened skills may be those with the most pressing need to access accurate information on child health; to navigate services, including family planning services; and to successfully use contraception. As a result, despite progress in expanding women’s educational attainment in LMICs, expectations about economic empowerment and declines in fertility—and other wide-ranging improvements—may be overstated.

There are important limitations to our study. Our measures of literacy and numeracy are fairly simple and do not assess important domains. For example, the Programme for the International Assessment of Adult Competencies (PIAAC) defines literacy as “understanding, evaluating, using and engaging with written texts to participate in society, to achieve one’s goals, and to develop one’s knowledge and potential” (OECD 2012:19). The ability to read two simple sentences does not capture the more advanced components of understanding and engaging with written texts, but it also does not capture more foundational literacy skills, such as identifying letters or reading simple words. Our numeracy measure perhaps captures basic numeracy skills more effectively but fails to capture more advanced skills, in accordance with the PIAAC definition of numeracy as “the ability to access, use, interpret and communicate mathematical information and ideas, in order to engage in and manage the mathematical demands of a range of situations in adult life” (OECD 2012:34). If we conceptualize literacy and numeracy on a continuum, the assessments included in our study capture a snapshot of skills at the lower end of that continuum. More nuanced assessments may provide additional insight into the types of skills that are lost or gained after childbearing as well as more direction for effective interventions.

Although comparing findings across three settings and four study samples provides rich insights into consistent patterns in the effects of childbearing on academic skills, we are unable to investigate the important contextual variations within each site in a way that may further elucidate our results. None of the study samples are nationally representative, so our results should not be interpreted as indicating variations between countries, although the patterns in each sample likely reflect country-level differences, in part. Last, because these three studies were designed and implemented separately, they did not measure all independent variables, including academic skills, in exactly the same way. It is even more striking, then, that the results remain consistent between settings.

## Conclusion

Our findings highlight the need for additional research on the effects of adolescent childbearing on academic skills, including assessing whether negative effects are sustained and whether interventions with adolescent mothers may counteract these effects. From a policy perspective, evidence of a threshold level of grade attainment, which appears to protect against skill loss, reinforces current efforts aimed at promoting universal secondary school completion (UNESCO 2016), although accelerated progress will be needed to achieve this goal in many settings (Psaki et al. 2017). Further, the vulnerability of academic skills among those with low levels of attainment likely reflects, in part, the poor quality of schooling in the study settings (Grant 2015; World Bank 2013, 2018). Improvements in school quality, leading to the development of stronger academic skills earlier in the school cycle, may also protect against skill loss.

Given the rapid transitions from school to motherhood experienced by many adolescent girls in LMICs, our findings demonstrate that the years after leaving school may be a critical period for skill retention or loss. Yet few existing policies and programs aim to secure and strengthen academic skills for young mothers. Programs that do target young mothers often focus on knowledge and use of services related to maternal and child health. In addition to continuing efforts to improve educational attainment and learning as an end in itself, given growing conflicts between childbearing and schooling, investments are also needed to bolster the skills of adolescent mothers in order to fulfill the longer-term demographic and economic promise of expanded access to schooling.

## Electronic supplementary material

ESM 1 (DOCX 44 kb)

## Footnotes

1 Although miscarriages have been used as natural experiments in studies estimating the causal effect of teenage childbearing on grade attainment, some researchers have argued that miscarriages are not always random events because those from more advantaged backgrounds may be more likely to have an abortion before a miscarriage occurs (Ashcraft and Lang 2006; Fletcher and Wolfe 2009; Hotz et al. 2005).

2 For each urban site, an additional four clusters were randomly selected as external controls from similar nearby areas. We exclude data from these external controls from our analyses because they were dropped in Round 5 of follow-up as a result of budgetary constraints.

3 The local language sentences were in Chichewa in Malawi; Nyanja (for all), and Bemba or Kaonde (by choice) in Zambia; and Bangla in Bangladesh.

4 The English sentences used were “Parents love their children” and “Children work hard in school.” The local language sentences were “Farming is hard work” and “The child is reading a book.” Respondents who were blind or visually impaired were excluded from analyses.

5 MIE is a parastatal organization that is charged by the Ministry of Education with curriculum development, assessment, and teacher training programs.

6 Grade 5 is the last year of primary school in Bangladesh, grade 7 is the last year of primary in Zambia, and grade 8 is the last year of primary in Malawi.

7 The timeframe for “current” work varies between study. In AGEP, this refers to work in the last seven days; in BALIKA, it refers to work in the last month; and in MSAS, it refers to work currently done “on a regular basis,” as defined by the respondent.

8 AGEP included 10- to 14-year-olds as well, but they are excluded from these analyses because they were not asked questions about childbearing and marriage.

9 We see this unexpected pattern for those with less than a grade 6 education in several models, although it is mostly not significant. This may reflect relatively uncommon patterns for girls in the study populations. For example, some evidence from Bangladesh indicates that girls from relatively better-off households may reach menarche at younger ages and drop out of school to marry. However, these same girls may also be stronger students and more able to improve their skills after childbearing than those who stay in school longer. This explanation is purely speculative, and the pattern may also be due to error given the small samples in this category.

10 As with English literacy, we again see indication of an unexpected positive effect of childbearing on local language literacy for those with less than grade 6 attainment in both the BALIKA and AGEP rural samples, but it is not significant in the BALIKA sample and is only borderline significant in the AGEP rural sample.

11 Given lower levels of literacy in Nyanja (the language of the first sentence in the local language assessment) compared with the local language in the other two studies, we ran the AGEP local language models using only the second sentence, which was in the language of each respondent’s choosing. We found that the results were consistent overall (available on request).

12 Until recently, Demographic and Health Surveys assumed that all respondents who had attended secondary school were literate; however, this has changed in the most recent (DHS-7) surveys (Croft et al. 2018).

13 Respondents were able to choose the language for the second sentence in the local language assessment. Following 2013 guidance from the Zambian Ministry of Education, Science, Vocational Training and Early Education, Zambian languages replaced English as the medium of instruction for grades 1–4; starting in grade 5, all students are taught in English. The Ministry declared that in grades 1–4 students should be taught in their “familiar” (or mother tongue) language, which varies by community. We do not have data on the language of instruction in grades 1–4 in the schools that AGEP respondents attended (UNICEF 2016).

14 This may also reflect different social roles if declines in cognitive function are purely due to lack of sleep.

## References

[CR1] Abadzi, H. (2003). *Adult literacy: A review of implementation experience* (World Bank OED Working Paper No. 29387). Washington, DC: Operations Evaluation Department, World Bank.

[CR2] Allison PD (2009). Fixed effects regression models.

[CR3] Amin S, Ainul S, Akter F, Alam MM, Hossain MI, Ahmed J, Rob U (2014). *From evidence to action: Results from the 2013 baseline survey for the BALIKA project* (Report).

[CR4] Ardington C, Menendez A, Mutevedzi T (2015). Early childbearing, human capital attainment and mortality risk: Evidence from a longitudinal demographic surveillance area in rural-KwaZulu-Natal, South Africa. Economic Development and Cultural Change.

[CR5] Ashcraft A, Lang K (2006). *The consequences of teenage childbearing* (NBER Working Paper No. 12485).

[CR6] Azevedo JP, Lopez-Calva LF, Perova E (2012). *Is the baby to blame? An inquiry into the consequences of early childbearing* (Policy Research Working Paper).

[CR7] Basu AM (2002). Why does education lead to lower fertility? A critical review of some of the possibilities. World Development.

[CR8] Behrman JA (2015). Does schooling affect women’s desired fertility? Evidence from Malawi, Uganda, and Ethiopia. Demography.

[CR9] Bledsoe C, Casterline J, Johnson-Kuhn J, Haaga J, National Research Council Commission on Behavioral and Social Sciences and Education, Committee on Population (1999). Critical perspectives on schooling and fertility in the developing world.

[CR10] Bongaarts J, Casterline J (2013). Fertility transition: Is sub-Saharan Africa different?. Population and Development Review.

[CR11] Breierova L, Duflo E (2003). *The impact of education on fertility and child mortality: Do fathers really matter less than mothers?* (Working Paper No. 217).

[CR12] Budig M, England P (2001). The wage penalty for motherhood. American Sociological Review.

[CR13] Casale D, Posel D (2011). English language proficiency and earnings in a developing country: The case of South Africa. Journal of Socio-Economics.

[CR14] Casey P (2000). A longitudinal study of cognitive performance during pregnancy and new motherhood. Archives of Women's Mental Health.

[CR15] Central Statistical Office (CSO) [Zambia], Ministry of Health (MOH) [Zambia], and ICF International (2014). Zambia Demographic and Health Survey 2013–14.

[CR16] Chall JS (1996). Stages of reading development.

[CR17] Chimombo, J., Kunje, D., Chimuzu, T., & Mchikoma, C. (2005). *The SACMEQ II project in Malawi: A study of the conditions of schooling and the quality of education* (Malawi working report, in SACMEQ Educational Policy Research Series). Harare, Zimbabwe: SACMEQ and the Ministry of Education, Malawi.

[CR18] Chowdhury, R., & Kabir, A. H. (2014). Language wars: English education policy and practice in Bangladesh. *Multilingual Education, 4*(21). 10.1186/s13616-014-0021-2

[CR19] Cleland J (2010). The benefits of educating women. Lancet.

[CR20] Cochrane SH (1979). Fertility and education: What do we really know?.

[CR21] Correll SJ, Benard S, Paik I (2007). Getting a job: Is there a motherhood penalty?. American Journal of Sociology.

[CR22] Crawley, R. A., Dennison, K., & Carter, C. (2003). Cognition in pregnancy and the first year post-partum. *Psychology and Psychotherapy: Theory, Research and Practice, 76,* 69–84. 10.1348/14760830260569265 12689436

[CR23] Croft TN, Marshall AMJ, Allen CK, DHS Program Staff (2018). Guide to DHS statistics.

[CR24] De Groot RHM, Vuurman EFPM, Hornstra G, Jolles J (2006). Differences in cognitive performance during pregnancy and early motherhood. Psychological Medicine.

[CR25] Diaz CJ, Fiel JE (2016). The effect(s) of teen pregnancy: Reconciling theory, methods, and findings. Demography.

[CR26] Durgunoğlu AY, Öney B, Kuşcul H (2003). Development and evaluation of an adult literacy program in Turkey. International Journal of Educational Development.

[CR27] Eloundou-Enyegue PM (2004). Pregnancy-related dropouts and gender inequality in education: A life-table approach and application to Cameroon. Demography.

[CR28] Fletcher JM, Wolfe BL (2009). Education and labor market consequences of teenage childbearing: Evidence using the timing of pregnancy outcomes and community fixed effects. Journal of Human Resources.

[CR29] Gakidou E, Cowling K, Lozano R, Murray CJL (2010). Increased educational attainment and its effect on child mortality in 175 countries between 1970 and 2009: A systematic analysis. Lancet.

[CR30] Geronimus AT, Korenman S (1992). The socioeconomic consequences of teen childbearing reconsidered. Quarterly Journal of Economics.

[CR31] Glewwe P (1999). Why does mother’s schooling raise child health in developing countries? Evidence from Morocco. Journal of Human Resources.

[CR32] Gorman KS, Pollitt E (1997). The contribution of schooling to literacy in Guatemala. International Review of Education.

[CR33] Grant MJ (2015). The demographic promise of expanded female education: Trends in the age at first birth in Malawi. Population and Development Review.

[CR34] Grant MJ, Hallman KK (2008). Pregnancy-related school dropout and prior school performance in KwaZulu-Natal, South Africa. Studies in Family Planning.

[CR35] Hartley MJ, Swanson EV (1986). *Retention of basic skills among dropouts from Egyptian primary schools* (Education and Training Series, Discussion Paper No. EDT40).

[CR36] Henry JD, Rendell PG (2007). A review of the impact of pregnancy on memory function. Journal of Clinical and Experimental Neuropsychology.

[CR37] Hewett PC, Austrian K, Soler-Hampejsek E, Behrman JR, Bozzani F, Jackson-Hachonda NA (2017). Cluster randomized evaluation of Adolescent Girls Empowerment Programme (AGEP): Study protocol. BMC Public Health.

[CR38] Hoekzema, E., Barba-Muller, E., Pozzobon, C., Picado, M., Lucco, F., Garcia-Garcia, D. . . . Vilarroya, O. (2017). Pregnancy leads to long-lasting changes in human brain structure. *Nature Neuroscience, 20,* 287–296. 10.1038/nn.4458 27991897

[CR39] Hotz VJ, McElroy SW, Sanders SW (2005). Teenage childbearing and its life cycle consequences: Exploiting a natural experiment. Journal of Human Resources.

[CR40] Jejeebhoy SJ (1995). Women’s education, autonomy, and reproductive behaviour: Experience from developing countries.

[CR41] Kane, J. B., Morgan, S. P., Harris, K. M., & Guilkey, D. K. (2013). The educational consequences of teen childbearing. *Demography, 50,* 2129–2150. 10.1007/s13524-013-0238-9 PMC3944136 24078155

[CR42] Kendall N (2007). Education for all meets political democratization: Free primary education and the neoliberalization of the Malawian school and state. Comparative Education Review.

[CR43] LeVine RA, LeVine S, Schnell-Anzola B, Rowe ML, Dexter E (2012). *Literacy and mothering: How women’s schooling changes the lives of the world’s children* (Child Development in Cultural Context series).

[CR44] Lloyd CB (2005). Growing up global: The changing transitions to adulthood in developing countries.

[CR45] Lloyd CB (2009). *New lessons: The power of educating adolescent girls* (Girls Count report).

[CR46] Lloyd CB, Mensch BS (2008). Marriage and childbirth as factors in dropping out from school: An analysis of DHS data from sub-Saharan Africa. Population Studies.

[CR47] Nguyen MC, Wodon Q (2012). *Global trends in child marriage* (Report).

[CR48] NIPORT (2016). Bangladesh Demographic and Health Survey 2014.

[CR49] NSO, & ICF (2017). *Malawi Demographic and Health Survey 2015–16* (Report). Zomba. Malawi: National Statistical Office.

[CR50] OECD (2012). Literacy, numeracy and problem solving in technology-rich environments: Framework for the OECD Survey of Adult Skills.

[CR51] Osili UO, Long BT (2008). Does female schooling reduce fertility? Evidence from Nigeria. Journal of Development Economics.

[CR52] Pritchett L (2013). The rebirth of education: Schooling ain’t learning.

[CR53] Psaki, S. R., Chuang, E. K., Melnikas, A. J., Wilson, D. B., & Mensch, B. S. (2019). Causal effects of education on sexual and reproductive health in low and middle-income countries: A systematic review and meta-analysis. *Social Science & Medicine: Population Health*. Advance online publication. 10.1016/j.ssmph.2019.100386 10.1016/j.ssmph.2019.100386 PMC6582211 31245525

[CR54] Psaki SR, McCarthy K, Mensch B (2017). Measuring gender equality in education: Lessons from trends in 43 countries. Population and Development Review.

[CR55] Ranchhod V, Lam D, Leibbrandt M, Marteleto L (2011). *Estimating the effect of adolescent fertility on educational attainment in Cape Town using a propensity score weighted regression* (SALDRU Working Paper No. 59).

[CR56] Santhya KG, Jejeebhoy SJ (2003). Sexual and reproductive health needs of married adolescent girls. Economic and Political Weekly.

[CR57] Smith-Greenaway E (2013). Maternal reading skills and child mortality in Nigeria: A reassessment of why education matters. Demography.

[CR58] Smith-Greenaway, E. (2017). Community context and child health: A human capital perspective. *Journal of Health and Social Behavior, 58,* 307–321. 10.1177/0022146517718897 29164944

[CR59] Soler-Hampejsek E, Mensch B, Psaki S, Grant M, Kelly C, Hewett P (2018). Reading and numeracy skills after school leaving in southern Malawi: A longitudinal analysis. International Journal of Educational Development.

[CR60] Stange K (2011). A longitudinal analysis of the relationship between fertility timing and schooling. Demography.

[CR61] Timaeus IM, Moultrie TA (2015). Teenage childbearing and educational attainment in South Africa. Studies in Family Planning.

[CR62] Trudell B (2016). Language choice and education quality in Eastern and Southern Africa: A review. Comparative Education.

[CR63] UNESCO (2007). *Mother tongue matters: Local language as a key to effective learning* (Report).

[CR64] UNESCO (2016). *Education for people and planet: Creating sustainable futures for all* (Global Education Monitoring Report).

[CR65] UNESCO Institute for Statistics (UIS) (2017). Bangladesh education and literacy data.

[CR66] Wagner, D. A. (1994). *Use it or lose it? The problem of adult literacy skill retention* (NCAL Technical Report, No. TR94-07). Philadelphia: National Center on Adult Literacy at University of Pennsylvania.

[CR67] Wagner DA, Spratt JE, Klein G, Ezzaki A (1989). The myth of literacy relapse: Literacy retention among fifth-grade Moroccan school leavers. International Journal of Educational Development.

[CR68] Webbink D, Martin NG, Visscher PM (2011). Does teenage childbearing reduce investment in human capital?. Journal of Population Economics.

[CR69] Weitzman A (2017). The effects of women’s education on maternal health: Evidence from Peru. Social Science & Medecine.

[CR70] World Bank (2013). Bangladesh education sector review—Seeding fertile ground: Education that works for Bangladesh.

[CR71] World Bank (2018). World development report 2018: Learning to realize education’s promise.

[CR72] World Bank Group (2016). Bangladesh: Country snapshot.

[CR73] World Bank, & UNICEF (2009). *Abolishing school fees in Africa: Lessons from Ethiopia, Ghana, Kenya, Malawi, and Mozambique* (Development Practice in Education report).

