# Supplementary material for: The Effects of Adolescent Childbearing on Literacy and Numeracy in Bangladesh, Malawi, and Zambia
Source: Demography. 2019 Sep 9;56(5):1899–929. doi: 10.1007/s13524-019-00816-z (PMC6797635; doi:10.1007/s13524-019-00816-z)
Supplement: Supplementary file 1 — (DOCX 44 kb) [file 13524_2019_816_MOESM1_ESM.docx]

**The effects of adolescent childbearing on literacy and numeracy in Bangladesh, Malawi, and Zambia**

Stephanie R. Psaki, Erica Soler-Hampejsek, Jyotirmoy Saha, Barbara S. Mensch, Sajeda Amin

**Online Appendix**

| **Table S1.** Study designs and samples | | | | | | | |  |
| --- | --- | --- | --- | --- | --- | --- | --- | --- |
| Study name | Country | Location | | Timeline | Sample | Follow-up Rate | Intervention | |
| Malawi Schooling and Adolescent Study (MSAS) | Malawi | Rural | Machinga, Balaka | Annually from 2007-2011, 2013  (6 years, 6 rounds) | 1337 girls | 89-91% of baseline sample interviewed in each round | None | |
| Bangladeshi Association for Life Skills, Income and Knowledge for Adolescents (BALIKA) | Bangladesh | Rural | Khulna, Narail, Satkhira | August 2013 (baseline); July 2015 (endline)  (2 years, 2 rounds) | 11609 girls total:   - 8721 - intervention - 2888 - control | 86% of baseline sample interviewed at endline | Four-arm cluster RCT designed to delay marriage | |
| Adolescent Girls Empowerment Program (AGEP) | Zambia | Rural | Central, Copperbelt, North-Western | Annually from 2013-2017  (4 years, 5 rounds) | 2273 girls total:   - 1708 - intervention - 565 - control | 83-90% of baseline sample interviewed in rounds 2-4; 80% of target sample interviewed in round 5* | Four-arm cluster RCT designed to improve health outcomes | |
|  |  | Urban | Central, Copperbelt, Lusaka |  | 2388 girls total:   - 1807 - intervention - 581 - control | 84-89% of baseline sample interviewed in rounds 2-4; 83% of target sample interviewed in round 5* |  |  |
| *Notes: We show the full female sample from each study. MSAS also included a sample of 1,312 males, who are excluded from these analyses due to the lower proportion of males experiencing a first reproductive event by the last round of the study (39% ever married, 34% ever had biological child), and to ensure comparability across studies. Study characteristics reflect only the MSAS female study sample. AGEP also included a sample of 574 girls from five external urban sites, who are excluded from these analyses as they were not followed up in round 5. *AGEP round 5 data was conducted with a reduced sample due to budgetary constraints.* | | | | | | | |  |

**Table S2.** Estimated effect of birth on standardized English oral reading score from linear fixed effects models and linear random effects models.

|  | BALIKA (Bangladesh) | | | | MSAS (Malawi) | | | | AGEP (Zambia) | | | | | | | |
| --- | --- | --- | --- | --- | --- | --- | --- | --- | --- | --- | --- | --- | --- | --- | --- | --- |
|  |  |  |  |  |  |  |  |  | Rural | | | | Urban | | | |
|  | Fixed Effects | | Random Effects | | Fixed Effects | | Random Effects | | Fixed Effects | | Random Effects | | Fixed Effects | | Random Effects | |
| Ever birth | 0.05 |  | 0.07 |  | 0.06 | † | 0.13 | * | 0.00 |  | 0.02 |  | -0.09 |  | -0.07 |  |
| Grade attainment |  |  |  |  |  |  |  |  |  |  |  |  |  |  |  |  |
| <6 grades | -0.79 | *** | -1.24 | *** | -0.76 | *** | -1.46 | *** | -0.82 | *** | -1.39 | *** | -0.66 | *** | -1.12 | *** |
| 6 grades | -0.37 | *** | -0.62 | *** | -0.20 | ** | -0.51 | *** | -0.60 | *** | -0.98 | *** | -0.38 | *** | -0.65 | *** |
| 7 grades | -0.26 | *** | -0.46 | *** | -0.06 | * | -0.25 | *** | -0.28 | *** | -0.52 | *** | -0.22 | *** | -0.40 | *** |
| 8 grades | -0.10 | *** | -0.22 | *** | -0.04 |  | -0.13 | *** | -0.07 | * | -0.18 | *** | -0.06 | * | -0.16 | *** |
| 9+ grades (ref) | -- |  | -- |  | -- |  | -- |  |  |  |  |  | -- |  |  |  |
| Time since school leaving |  |  |  |  |  |  |  |  |  |  |  |  |  |  |  |  |
| Currently attending school (ref) | -- |  | -- |  | -- |  | -- |  | -- |  |  |  | -- |  |  |  |
| <1 year ago/ dropped after baseline | -0.11 | *** | -0.33 | *** | -0.05 | † | -0.08 | * | -0.05 | † | -0.13 | *** | 0.10 | ** | -0.01 |  |
| 1-2 years ago | -- |  | -- |  | -0.09 | * | -0.11 | * | -0.05 |  | -0.17 | *** | 0.03 |  | -0.13 | ** |
| 2+ years ago | -- |  | -- |  | -0.28 | *** | -0.22 | *** | 0.02 |  | -0.13 | ** | 0.15 | ** | -0.04 |  |
| Currently working | 0.050 | † | 0.08 | *** | 0.01 |  | 0.02 |  | -0.01 |  | -0.01 |  | -0.07 | * | -0.07 | ** |
| Household wealth | 0.03 | *** | 0.08 | *** | 0.00 |  | 0.00 |  | 0.01 |  | 0.01 |  | -0.01 |  | 0.00 |  |
| Ever birth * grade attainment |  |  |  |  |  |  |  |  |  |  |  |  |  |  |  |  |
| Birth* <6 grades | -0.05 |  | -0.05 |  | -0.27 | * | -0.43 | *** | 0.38 |  | 0.16 |  | -0.28 | * | -0.63 | *** |
| Birth*6 grades | -0.55 | *** | -0.59 | *** | -0.34 | ** | -0.41 | *** | -0.35 | † | -0.50 | ** | -0.13 |  | -0.30 |  |
| Birth*7 grades | -0.27 | * | -0.42 | *** | -0.19 | * | -0.24 | *** | -0.22 | * | -0.34 | *** | 0.14 |  | 0.05 |  |
| Birth*8 grades | -0.24 | * | -0.33 | *** | 0.00 |  | -0.01 |  | -0.11 |  | -0.16 |  | 0.06 |  | 0.03 |  |
| Birth*9+ grades | -- |  | -- |  | -- |  | -- |  |  |  |  |  |  |  |  |  |
| Age | -- |  | -0.02 | ** | -- |  | -0.04 | *** | -- |  | 0.05 | ** |  |  | -0.02 |  |
| Program Assignment/Exposure | -- |  | 0.01 |  | -- |  | -- |  | 0.00 | * | 0.00 |  | 0.00 |  | 0.00 |  |
| Constant | 0.12 | ** | 0.31 | ** | 3.71 |  | 4.50 | *** | 0.24 | *** | -0.45 |  | 0.30 | *** | 0.67 | † |
| Number of observations | 15276 | | 15276 | | 4776 | | 4776 | | 3066 | | 3066 | | 3157 | | 3157 | |
| Number of groups (subjects) | 7638 | | 7638 | | 856 | | 856 | | 697 | | 697 | | 706 | | 706 | |
| Hausman test | p<0.0001 | | | | p<0.0001 | | | | p<0.0001 | | | | p<0.0001 | | | |
| *Notes: Oral reading score ranges from 0 to 4 (for each sentence, 0 = cannot read at all, 1=can read partial sentence, 2=can read full sentence; scores on each sentence are summed). All models adjust for geographic clustering. BALIKA models use inverse probability weighting to account for loss to follow-up at endline. Since BALIKA only included two rounds of data collection, time since school leaving is dichotomous, coded as either in school or out of school at endline. Hausman tests compare fixed effects and random effects models without weighting or clustering, so results may differ slightly from those reported in other tables. ***p<0.001; **p<0.01; *p<0.05* | | | | | | | | | | | | | | | | |

**Table S3.** Estimated effect of birth on standardized local language oral reading score from linear fixed effects models and linear random effects models.

|  | BALIKA (Bangladesh) | | | | MSAS (Malawi) | | | | AGEP (Zambia) | | | | | | | |
| --- | --- | --- | --- | --- | --- | --- | --- | --- | --- | --- | --- | --- | --- | --- | --- | --- |
|  |  |  |  |  |  |  |  |  | Rural | | | | Urban | | | |
|  | Fixed Effects | | Random Effects | | Fixed Effects | | Random Effects | | Fixed Effects | | Random Effects | | Fixed Effects | | Random Effects | |
| Ever birth | 0.02 |  | 0.08 | * | 0.00 |  | 0.03 |  | 0.09 | † | 0.06 |  | 0.02 |  | 0.02 |  |
| Grade attainment |  |  |  |  |  |  |  |  |  |  |  |  |  |  |  |  |
| <6 grades | -0.46 | *** | -0.85 | *** | -0.09 | † | -0.28 | *** | -0.69 | *** | -1.08 | *** | -0.65 | *** | -0.93 | *** |
| 6 grades | -0.07 | * | -0.19 | *** | 0.01 |  | -0.05 |  | -0.58 | *** | -0.83 | *** | -0.38 | *** | -0.58 | *** |
| 7 grades | -0.06 | * | -0.12 | *** | 0.00 |  | -0.04 |  | -0.31 | *** | -0.48 | *** | -0.28 | *** | -0.42 | *** |
| 8 grades | -0.02 |  | -0.06 | ** | 0.01 |  | 0.00 |  | -0.11 | ** | -0.20 | *** | -0.12 | ** | -0.20 | *** |
| 9+ grades (ref) | -- |  | -- |  | -- |  | -- |  | -- |  | -- |  | -- |  | -- |  |
| Time since school leaving |  |  |  |  |  |  |  |  |  |  |  |  |  |  |  |  |
| Currently attending school (ref) | -- |  | -- |  | -- |  | -- |  | -- |  | -- |  | -- |  | -- |  |
| <1 year ago/ dropped after baseline | 0.06 | † | -0.11 | *** | 0.01 |  | -0.02 |  | -0.04 |  | -0.09 | ** | 0.00 |  | -0.08 | * |
| 1-2 years ago | -- |  | -- |  | 0.00 |  | -0.03 |  | -0.06 | † | -0.13 | ** | -0.04 |  | -0.15 | ** |
| 2+ years ago | -- |  | -- |  | -0.03 |  | -0.06 |  | -0.06 |  | -0.14 | ** | 0.05 |  | -0.08 |  |
| Currently working | -0.06 | † | -0.02 |  | 0.00 |  | 0.00 |  | 0.03 |  | 0.04 |  | -0.06 | † | -0.05 |  |
| Household wealth | 0.01 |  | 0.02 | *** | 0.00 |  | 0.00 |  | 0.00 |  | 0.00 |  | -0.01 |  | -0.01 |  |
| Ever birth * grade attainment |  |  |  |  |  |  |  |  |  |  |  |  |  |  |  |  |
| Birth* <6 grades | 0.12 |  | -0.33 |  | -0.05 |  | -0.12 | * | 0.37 | † | 0.27 |  | -0.15 | * | -0.25 | ** |
| Birth*6 grades | -0.42 | † | -0.30 |  | -0.03 |  | -0.06 |  | -0.01 |  | -0.13 |  | -0.05 |  | -0.09 |  |
| Birth*7 grades | -0.30 |  | -0.29 | * | 0.00 |  | -0.02 |  | -0.25 | * | -0.26 | * | -0.03 |  | -0.05 |  |
| Birth*8 grades | 0.05 |  | 0.02 |  | -0.01 |  | -0.02 |  | -0.05 |  | -0.08 |  | -0.02 |  | -0.03 |  |
| Birth*9+ grades (ref) | -- |  | -- |  | -- |  | -- |  |  |  |  |  |  |  |  |  |
| Age | -- |  | -0.01 | * | -- |  | 0.00 |  | -- |  | 0.05 | * | -- |  | 0.01 |  |
| Program Assignment/Exposure | -- |  | -0.02 |  | -- |  | -- |  | 0.00 | † | 0.00 |  | 0.00 | * | 0.00 | † |
| Constant | 0.05 |  | 0.26 | *** | 3.88 | *** | 3.95 | *** | 0.28 | *** | -0.36 |  | 0.32 | *** | 0.31 |  |
| Number of observations | 15276 | | 15276 | | 4776 | | 4776 | | 3066 | | 3066 | | 3157 | | 3157 | |
| Number of groups (subjects) | 7638 | | 7638 | | 856 | | 856 | | 697 | | 697 | | 706 | | 706 | |
| Hausman test | p<0.0001 | | | | p<0.0001 | | | | p<0.0001 | | | | p<0.0001 | | | |
| *Notes: Oral reading score ranges from 0 to 4 (for each sentence, 0 = cannot read at all, 1=can read partial sentence, 2=can read full sentence; scores on each sentence are summed). All models adjust for geographic clustering. BALIKA models use inverse probability weighting to account for loss to follow-up at endline. Since BALIKA only included two rounds of data collection, time since school leaving is dichotomous, coded as either in school or out of school at endline. (a) model fails to meet the asymptotic assumptions of the Hausman test.*  ****p<0.001; **p<0.01; *p<0.05; †p<0.10* | | | | | | | | | | | | | | | | |

**Table S4.** Estimated effect of birth on standardized numeracy score from linear fixed effects models and linear random effects models.

|  | BALIKA (Bangladesh) | | | | MSAS (Malawi) | | | |
| --- | --- | --- | --- | --- | --- | --- | --- | --- |
|  | Fixed Effects | | Random Effects | | Fixed Effects | | Random Effects | |
| Ever birth | 0.02 |  | 0.10 | * | -0.17 | † | 0.03 |  |
| Grade attainment |  |  |  |  |  |  |  |  |
| <6 grades | -0.47 | *** | -0.89 | *** | -0.31 |  | -2.15 | *** |
| 6 grades | -0.26 | *** | -0.48 | *** | -0.20 | † | -1.31 | *** |
| 7 grades | -0.18 | *** | -0.37 | *** | 0.15 | † | -0.56 | *** |
| 8 grades | -0.11 | *** | -0.22 | *** | 0.23 | *** | -0.14 | † |
| 9+ grades (ref) | -- |  | -- |  | -- |  | -- |  |
| Time since school leaving |  |  |  |  |  |  |  |  |
| Currently attending school (ref) | -- |  | -- |  | -- |  | -- |  |
| <1 year ago/ dropped after baseline | 0.08 | * | -0.21 | *** | -0.32 | *** | -0.25 | ** |
| 1-2 years ago | -- |  |  |  | -0.23 | ** | -0.07 |  |
| 2+ years ago | -- |  |  |  | -0.34 | ** | 0.14 |  |
| Currently working | 0.06 | * | 0.14 | *** | -0.04 |  | 0.01 |  |
| Household wealth | 0.03 | ** | 0.08 | *** | 0.00 |  | -0.01 |  |
| Ever birth * grade attainment |  |  |  |  |  |  |  |  |
| Birth* <6 grades | -0.21 |  | -0.26 | † | -0.39 | * | -0.68 | *** |
| Birth*6 grades | -0.24 |  | -0.31 | † | -0.05 |  | -0.23 |  |
| Birth*7 grades | -0.20 |  | -0.31 | * | -0.37 | ** | -0.50 | ** |
| Birth*8 grades | -0.04 |  | -0.18 | * | -0.26 | * | -0.32 | ** |
| Birth*9+ grades | -- |  | -- |  | -- |  | -- |  |
| Age | 0.08 |  | -0.04 | *** | -- |  | -0.16 | *** |
| Program Assignment | -- |  | 0.01 |  | -- |  | -- |  |
| Constant | 0.08 |  | 0.58 | *** | 6.07 | *** | 9.31 | *** |
| Number of observations | 15276 | | 15276 | | 4789 |  | 4789 |  |
| Number of groups (subjects) | 7638 | | 7638 | | 856 |  | 856 |  |
| Hausman test | p<0.0001 | | | | p<0.0001 (a) | | | |
| *Notes: Oral reading score ranges from 0 to 4 (for each sentence, 0 = cannot read at all, 1=can read partial sentence, 2=can read full sentence; scores on each sentence are summed). All models adjust for geographic clustering. BALIKA models use inverse probability weighting to account for loss to follow-up at endline. For BALIKA and AGEP, only included two rounds of data collection are available for these analyses. Therefore, time since school leaving is dichotomous, coded as either in school or out of school at the follow-up round. (a) model fails to meet the asymptotic assumptions of the Hausman test. ***p<0.001; **p<0.01; *p<0.05; †p<0.10* | | | | | | | | |

| **Table S5.** Selection of questions in each study included in current numeracy analyses. | | |
| --- | --- | --- |
|  | MSAS (Malawi) | BALIKA (Bangladesh) |
| Fill in the blank | Write the missing number: 14, 15, __, 17, __, 19, 20, __, __, 23 | |
| Subtraction | Write these numbers in order starting from the biggest number to the smallest: 8, 10, 5, 11, 4 | |
| Addition | 137+56 | 137+57 |
| Subtraction | 18-7 | 18-7 |
| Multiplication | 23x3 | 23x12 |
| Division | 459/3 | 459/3 |
| Word problem 1 | Each banana costs 50 tambala. How much would 3 bananas cost? | Each banana costs 8 taka. How much would 3 bananas cost? |
| Word problem 2 | You have 75 tambala and you want to buy a pencil that costs 67 tambala. How much change would you get? | You have 75 taka and you want to buy a pen that costs 67 taka. How much change would you get? |
| *Note:* The assessment was conducted in Chichewa in MSAS (Malawi) and in Bangla in BALIKA (Bangladesh). | | |
